# Brewer’s Spent Grain Flour: Chemical Composition, Functional Properties, and Influence on Gut Microbiota

**DOI:** 10.3390/foods15111931

**Published:** 2026-05-29

**Authors:** Cristina Clavel, Vanesa Núñez-Gómez, Nieves Baenas, Rocío González-Barrio, Belén Olga Ferrando, Lorena Sánchez-Martínez, Marina Santaella, María Jesús Periago

**Affiliations:** 1 Department of Food Science and Nutrition, Faculty of Veterinary Sciences, University of Murcia, Campus of International Excellence “Campus Mare Nostrum”, Campus of Espinardo, Espinardo, 30.100 Murcia, Spain; cclavelc@estrellalevante.es (C.C.); vanesa.nunez@um.es (V.N.-G.); nieves.baenas@um.es (N.B.); rgbarrio@um.es (R.G.-B.); obelen.ferrando@um.es (B.O.F.); lorena.sanchez14@um.es (L.S.-M.); mjperi@um.es (M.J.P.); 2 Estrella de Levante Fábrica de Cervezas S.A.U., C. Mayor, 171, Espinardo, 30.100 Murcia, Spain

**Keywords:** brewer’s spent grain, dietary fibre, in vitro fermentation, gut microbiota, short-chain fatty acids, (poly)phenols, functional ingredients, by-products

## Abstract

Brewers’ spent grain (BSG), which accounts for approximately 85% of the by-products generated during beer production, is a valuable source of dietary fibre, proteins and antioxidant compounds. This study aimed to characterise the chemical composition, techno-functional properties, antioxidant capacity and potential prebiotic effect of BSG flour as a sustainable functional ingredient. Dietary fibre composition, mineral content, and extractable and non-extractable (poly)phenol fractions were determined. The prebiotic potential of BSG flour was evaluated using an in vitro fermentation model with human faeces. Microbial metabolic activity was assessed through the production of short-chain fatty acids (SCFAs), lactate and ammonium, alongside changes in antioxidant capacity during fermentation, while microbiota composition was analysed by 16S rRNA amplicon sequencing. BSG flour showed high levels of insoluble fibre, mainly hemicellulose and arabinoxylans, as well as proteins and non-extractable (poly)phenols, particularly hydroxycinnamic acid derivatives. In vitro fermentation led to a significant increase in SCFA production, particularly acetate and propionate, indicating active degradation of fibre polysaccharides. These metabolic changes were accompanied by enhanced antioxidant capacity and shifts in microbiota composition, including an increased relative abundance of Bifidobacterium species. Overall, this study suggests that BSG flour could be used as a novel ingredient for the development of dietary-fibre-rich foods with potential gut health benefits.

## 1. Introduction

Beer is the most consumed alcoholic beverage in the world, with its global production reaching 1.88 billion hectolitres in 2023 [1]. Beer is the most consumed alcoholic beverage in the world, with its global production reaching 1.88 billion hectolitres in 2023 [1]. The brewing industry generates a large amount of by-products from the different steps of the industrial process; these by-products include second barley, rootlets, barley powder, wet yeast and brewer’s spent grain (BSG). Brewer’s spent grain, also known as bagasse, is the solid remains of malt, hops and adjuncts like rice and corn, which are retained after filtering the beer wort.

BSG accounts for about 85% of the total by-products generated during beer manufacture, with around 20 kg of bagasse produced per hectolitre of beer [2]. Storage of BSG is challenging, mainly due to the large quantities generated and its high moisture content, which make it highly perishable and prone to microbiological spoilage; for this reason, it is most used as animal feed [3]. Proposed industrial uses for BSG include composting, the production of biogas and bioethanol, use as a substrate for enzyme production, use as an absorbent material, and applications in concrete, ceramic materials, and paper production [4].

Nowadays, one of the main challenges for the sustainable food industry is achieving material circularity and the reuse and recycling of natural resources to reduce environmental impact and even generate economic compensation for industries [5]. Agri-food by-products still contain many nutrients and are particularly rich in lignocellulosic materials, which are part of dietary fibre, and in bioactive compounds, such as (poly)phenols, carotenoids, glucosinolates, etc., which can be used in other applications [6]. The by-products from the brewing industry contain large amounts of dietary fibre and proteins trapped in their matrix, so finding applications for BSG, a fibre-rich ingredient, in the food industry would be an opportunity for brewing companies to implement circular economy practices.

Dietary fibre is considered an important nutrient, and its consumption has been associated with the prevention of non-communicable diseases, such as cardiovascular diseases, diabetes, obesity, and colon cancer. An adequate intake of dietary fibre positively influences metabolic processes by reducing the absorption of glucose and cholesterol during digestion and exerting a prebiotic effect when undigestible polysaccharides reach the colon. Cellulose, hemicellulose, and pectins are the main polysaccharides in fibre and are partially or completely degraded by colonic bacteria, producing short-chain fatty acids (SCFAs), such as acetic, propionic, and butyric acids, which are known to maintain host intestinal and metabolic health [7]. In addition, BSG also contains (poly)phenolic compounds, secondary metabolites that have been shown to act as natural antioxidants and anti-inflammatory agents in cell cultures [8]. Due to their chemical complexity and high molecular weight, or because these antioxidants are bound to cell wall polysaccharides, (poly)phenols are poorly absorbed by the small intestine (around 5–10% of the ingested amount is absorbed), reaching the colon with minimal degradation by digestive enzymes. In the colon, they are released after the gut microbiota ferment non-starch polysaccharides, accumulating in the intestinal lumen. They can then be metabolized by the gut microbiota into bioavailable, low-molecular-weight phenolic metabolites, which help maintain gut health [9].

The combined effects of the dietary fibre and (poly)phenols in BSG have not been extensively studied. Therefore, this study aimed to characterise the chemical composition, techno-functional properties, antioxidant capacity, and prebiotic potential of BSG flour. Using an in vitro fermentation model with human faecal microbiota, we evaluated BSG flour’s impact on gut health. The findings will help determine the suitability of BSG as an ingredient for developing fibre-rich food products with potential health benefits.

## 2. Materials and Methods

### 2.1. Samples

Nine batches of BSG samples were collected after filtering beer during an industrial process. The BSG samples were provided by the Estrella de Levante Fábrica de Cervezas S.A.U. brewery (Murcia, Spain) and were frozen and stored at −18 °C until analysis. These samples were obtained from light-colored Pilsen malt, which is lightly roasted and used to brew lager-type beer, characterized as a clear and refreshing beer. To obtain BSG flour, the BSG samples were dried in an oven for 48 h at 80 °C and then ground in a Thermomix^®^ (model TM31; Vorwerk, Wuppertal, Germany). The resulting flour was then passed through a 25-mesh sieve to obtain a more homogeneous particle-size fraction (100% of particles smaller than 710 µm). The samples were stored at 4 °C until the analyses were carried out.

### 2.2. Proximate BSG and BSG Flour Compositions

The proximate compositions of the BSG and BSG flour were determined using the AOAC methods [10]. The protein content was determined using the Kjeldahl method with a Kjeltec System 2100 (Tecator, Höganas, Sweden). The total fat content was quantified by gravimetric extraction using a Soxhlet Avanti 2055 (Tecator, Sweden). The ash content was determined after incinerating the samples at 525 °C for 24 h in a muffle furnace. The total dietary fibre (TDF), soluble fibre (SDF) and insoluble fibre (IDF) contents were determined using the enzymatic-gravimetric method described by Prosky [11] and a Fibertec System E-1023 (Tecator, Sweden). The total carbohydrate content was calculated as the difference between the moisture, protein, fat, and ash contents, and the caloric value or total energy was estimated using the WHO conversion factors (proteins: 4 kcal/g; carbohydrates: 4 kcal/g; fats: 9 kcal/g). The proximate composition was expressed as a percentage of the fresh weight for the BSG and of the dry weight for the BSG flour; the total energy is presented as kcal/100 g.

### 2.3. Extraction and Quantification of the Extractable and Non-Extractable (Poly)phenolic Compounds

The extractable (poly)phenolic compounds (EPPs) and non-extractable (poly)phenolic compounds (NEPPs) in BSG and BSG flour were quantified using the method of Arranz et al. [12] with modifications [13]. For EPP extraction, 5 mL of a methanol/water/formic acid mixture (79:20:1, *v*/*v*) was added to 300 mg of BSG flour. The mixture was stirred and centrifuged at 4500 *g* for 10 min at room temperature. The supernatant was evaporated under vacuum at 35 °C using a Laborota-4002 rotary evaporator (Heidolph, Schwabach, Germany). The residue was reconstituted in 10 mL of Milli-Q water acidified with 0.1% formic acid. The sample was then loaded onto a pre-conditioned Sep-Pak C18 cartridge (Waters Corporation, Milford, MA, USA). After washing the cartridge with 10 mL of Milli-Q water, the EPPs were eluted with 1 mL of methanol. To extract the NEPPs, the remaining pellet was hydrolyzed with a methanol/sulfuric acid solution (9:1, *v*/*v*, ≈1.84 M) and incubated in a shaking incubator (VorTemp 1550, LabNet Biotécnica, Madrid, Spain) at 85 °C for 20 h. The sample was then centrifuged, processed as described for the EPPs, and eluted with methanol. The (poly)phenolic compounds in the EPP and NEPP fractions were quantified using the Folin–Ciocalteu colorimetric method [14], adapted for a Synergy microplate spectrophotometer (BioTek Instruments, Winooski, VT, USA) by measuring absorbance at 750 nm. Gallic acid (Riedel-de Haën, Hanover, Germany) served as the standard, and results were expressed as milligrams of gallic acid equivalent (GAE) per gram. The total (poly)phenolic content was calculated as the sum of the EPP and NEPP fractions and expressed in the same units.

### 2.4. Amino Acid Content and Mineral Composition of BSG Flour

The free and bound amino acids were analyzed by ultra-performance liquid chromatography–tandem mass spectrometry (UPLC-MS/MS) [15]. Results were expressed in mg/g, and amino acids were classified as essential or non-essential. In addition, the percentage of the total protein content of the sample for each amino acid was calculated. The mineral content of the BSG flour was analyzed by inductively coupled plasma optical emission spectrometry (ICP-OES) according to the AOAC method 985.35. Results were expressed as mg/100 g, and the dietary reference value (DRV) met by 100 g of the sample was included. Both analyses were carried out using the analytical services of CEBAS-CSIC (Murcia, Spain).

### 2.5. Neutral Sugars and Uronic Acids in Dietary Fibre in BSG Flour

The chemical composition of the dietary fibre in the BSG flour was assessed by quantifying its neutral sugar content using gas–liquid chromatography and its uronic acid content spectrophotometrically, as described by Englyst et al. [16].

First, the sample was digested in vitro using the procedure described for dietary fibre analysis, and the fibre residue was precipitated with ethanol. The residue containing indigestible non-starch polysaccharides was hydrolysed with 12 M H_2_SO_4_ at 35 °C for 1 h to disperse the cellulose. The acid was diluted to approximately 2 M with distilled water, and the samples were heated at 100 °C for 1 h to complete the hydrolysis. After acid hydrolysis, the samples were placed in an ice–water bath and neutralised by the addition of 12.5 M ammonia solution until an alkaline pH was reached. The pH was checked to ensure complete neutralisation, and additional ammonia was added if necessary.

A 1 mL aliquot of the hydrolysed sample was subjected to derivatisation to produce alditol acetate derivatives of neutral sugars. These derivatives were analysed using gas–liquid chromatography on an Agilent 7890B system (Mechelen, Germany) equipped with a flame ionisation detector (FID) and a DB-225 capillary column (Supelco, Bellefonte, PA, USA). The chromatographic conditions were as follows: the injector was set to 280 °C; the oven was programmed to hold the temperature at 210 °C for 5 min and then increase it by 5 °C/min to 240 °C, which was maintained for 9 min; and the detector was maintained at 280 °C. Commercial standards (Sigma-Aldrich, St. Louis, MO, USA) were used to identify the peaks in the chromatograms and the area under each peak was recorded to calculate the proportions of each sugar (fucose, xylose, mannose, rhamnose, arabinose, galactose and glucose). In addition, the hydrolysed residues were used to quantify uronic acids using the colorimetric method of Scott [17]. Briefly, the sample was mixed with H_2_SO_4_ and incubated for 40 min at 70 °C in the presence of 2% NaCl and 3% H_3_BO_3_. This reaction produces 5-formyl-2-furancarboxylic acid, which selectively reacts with 3-5-dimethylphenol. The uronic acid content was expressed as a percentage by measuring absorbance at 400 and 450 nm, using galacturonic acid (Sigma-Aldrich, St. Louis, MO, USA) as the standard. Finally, taking into consideration the neutral sugar and uronic acid contents, the proportions of polysaccharides (pectin, hemicellulose and cellulose) in the BSG flour dietary fibre were estimated using the following formulas [18].

Cellulose = glucose × 0.9

Hemicellulose = fucose + xylose + mannose + (glucose × 0.1)

Pectin = rhamnose + arabinose + galactose + uronic acid

### 2.6. Techno-Functional Properties of BSG Flour

Fat absorption capacity (FAC), hydration properties—swelling capacity (SWC) and water retention capacity (WRC)—osmotic pressure (OP), and glucose diffusion retardation index (GDRI) were evaluated to characterize the techno-functional properties of the BSG flour. FAC, which indicates the amount of fat absorbed by the sample, was determined according to the method described by Sosulski et al. [19]. For hydration properties, SWC and WRC, which measure the flour’s water-retention ability and reflect its viscosity, were assessed using the method described in the scientific literature [20]. Osmotic pressure was determined using a Knauer osmometer (Berlin, Germany) to assess the physiological effects on the gastrointestinal tract after ingestion of the fibre-rich BSG flour. The GDRI was assessed using an in vitro diffusion method in which the sample was mixed with a glucose solution [21]. The amount of diffused glucose was quantified using an oxidase enzymatic colorimetric method (GOD-PAP method, Boehringer-Mannheim) after 15 and 30 min.

### 2.7. Analysis of EPPs and NEPPs in BSG Flour Using HPLC-DAD

The individual (poly)phenolic compounds in the EPP and NEPP fractions from BSG flour were identified and quantified using the method described by González-Barrio et al. [22]. An HPLC 1200 series system equipped with a diode array detector (DAD) (Agilent Technologies, Waldbronn, Germany) was used, scanning wavelengths from 200 to 600 nm. Separation of the (poly)phenols was performed using a LiChroCART RP-18 column (250 × 4.6 mm, i.d. 5 μm) paired with a pre-column (4 × 4 mm) of the same material (Merck, Darmstadt, Germany). The mobile phases consisted of 1% aqueous formic acid (solvent A) and acetonitrile (solvent B), delivered at a flow rate of 1 mL/min. The elution process started with a linear gradient from 2% to 40% solvent B over 50 min, followed by a wash step and a return to the initial conditions. Identification of individual (poly)phenols in the samples was based on their UV spectra and retention times, as reported by González-Barrio et al. [22]. Hydroxycinnamic acid derivatives were quantified from their chromatographic peak areas recorded at 320 nm and expressed as chlorogenic acid equivalents. Flavonol conjugates were quantified based on their peak areas at 360 nm as quercetin-3-O-rutinoside equivalents. Both standards were purchased from Sigma-Aldrich (St. Louis, MO, USA).

### 2.8. Antioxidant Capacity of BSG Flour

The antioxidant capacity of the EPP and NEPP extracts from the BSG flour was analysed using the ferric reducing antioxidant power (FRAP) method [23]. The FRAP method was adapted for a microplate spectrophotometer (BioTek Instruments, Winooski, VT, USA), and the results were expressed as μmol of Trolox equivalents (TE)/g, using Trolox solutions as the standards (Sigma-Aldrich, St. Louis, MO, USA). The total antioxidant capacity was determined as the sum of the FRAP values from the EPP and NEPP fractions.

### 2.9. Prebiotic Effect of BSG Flour

The prebiotic effect of the BSG flour was evaluated by in vitro fermentation of a human fecal slurry that had been pretreated with an in vitro digestion process. First, the BSG flour was subjected to enzymatic digestion to simulate gastrointestinal conditions according to the standardised INFOGEST I protocol [24]. After digestion, the samples were lyophilised and used as substrates for in vitro colonic fermentation.

Fermentation was performed using pooled faecal samples from eight healthy, non-smoking, non-drinking female volunteers aged 30 to 40 years. All participants followed a varied Mediterranean diet, and none had taken antibiotics in the three months before sample collection. Volunteers were screened to confirm the absence of diagnosed pathologies or gastrointestinal disorders. The study protocol, including the recruitment process, was approved by the Research Ethics Committee (CEI code: 3484/2021) and the Experimental Biosafety Committee (CBE code: 412/2021) of the University of Murcia. All participants provided written informed consent before participation, and there were no dropouts during the study. They received materials for sample collection and were instructed on the procedure.

Faecal samples were collected under anaerobic conditions two hours before the start of the experiment. Upon arrival at the lab, a pooled faecal suspension was prepared by combining 12 g of fresh faeces from each volunteer, following the method of Aguirre et al. [25]. The faecal material was homogenized in a phosphate-buffered solution to produce a 32% suspension. Five milliliters of this suspension was added to McCartney fermentation bottles containing 44 mL of fermentation medium prepared with 0.5% (*w*/*v*) tryptone and micromineral and macromineral solutions, as described by González-Barrio et al. [26]. The pH was adjusted to 7 using 6M HCl, and 1 mL of 1% resazurin solution (*w*/*v*) was added as a redox indicator. Two hundred mg of freezer-dried, digested BSG meal was added to the fermentation bottles. Positive control bottles (C+) contained fermentation medium, the faecal suspension, and glucose, whereas negative control bottles (C−) contained only fermentation medium and the faecal suspension without any carbon substrate. The controls were compared with the experimental bottles to assess the viability of the faecal microbiota and to determine any effect of the substrate on the faecal suspension. The bottles were purged with nitrogen to remove oxygen, indicated by a color change from blue to pink, confirming anaerobic conditions. The fermentation flasks were then incubated at 37 °C in a shaking water bath at 60 strokes/min for 48 h. Gas production and pH were monitored throughout the fermentation process, and aliquots of the fermentation medium were collected at 0, 4, 8, 24, and 48 h using a sterile syringe to measure changes in SCFA, ammonium, and lactic acid levels, as well as antioxidant capacity, which reflects the metabolic activity of the microbiota. The samples were immediately stored at −80 °C until analysis. Fermentation of the BSG sample and control samples (C+ and C−) was performed in triplicate.

### 2.10. Analysis of SCFAs Using GC-FID

Aliquots of the faecal slurry collected after 0, 4, 8, 24 and 48 h of fermentation were analysed for the production of SCFAs, including acetic, propionic, butyric, isobutyric, valeric, isovaleric, caproic, isocaproic and heptanoic acids. The analysis was performed using gas–liquid chromatography with flame ionisation detection (GC-FID) according to the method described by Anson et al. [27], with the modifications used by Baenas et al. [28]. The faecal suspensions were mixed for 5 min with a solution consisting of 20% formic acid/water, methanol and 2-ethylbutyric acid (used as the internal standard at a concentration of 2 mg/mL in methanol) in a ratio of 1:4.5:1 (*v*/*v*/*v*). After mixing, the samples were centrifuged at 16,110 *g* for 15 min at room temperature. The resulting supernatants were filtered through 0.22 µm PTFE filters (Ø 13 mm; VWR International, Radnor, PA, USA) and analysed using GC-FID.

The chromatographic analysis was performed on an Agilent GC system equipped with a flame ionization detector (FID) and a 7683B autosampler (Agilent Technologies, Santa Clara, CA, USA). A fused-silica Nukol™ capillary column (30 m × 0.25 mm I.D., 0.25 µm film thickness; Supelco, USA) was used to separate the SCFAs. Helium was used as the carrier gas at a flow rate of 25 mL/min. The oven temperature was initially set at 80 °C and held for 5 min, then increased to 185 °C at 5 °C/min. Two microliters (2 μL) of the sample were injected in splitless mode, with the injection port maintained at 220 °C. Hydrogen and air, used as the make-up gases, flowed at 30 mL/min and 400 mL/min, respectively. The FID was operated at 220 °C, and each analysis took 26 min. SCFAs were identified by comparing their retention times with those of a standard SCFA mixture (Supelco, USA). The concentrations of individual SCFAs were reported in millimolar (mM).

### 2.11. Analysis of Ammonium and Lactate Production and Antioxidant Capacity During Fermentation

Ammonium and lactate production were analysed to evaluate microbial activity after 0, 4, 8, 24, and 48 h of in vitro fermentation. The ammonium analysis method was adapted from the total volatile base nitrogen determination [29]. The sample was distilled for 4 min at basic pH, and ammonia was collected in a flask containing 25 mL of a 3% boric acid solution (Panreac, Barcelona, Spain). Finally, the nitrogen content was quantified by neutralisation titration with 0.01 N HCl (Panreac, Barcelona, Spain). The total ammonium content, expressed as mg of ammonium/mL, was calculated using the following equation: mg ammonium/mL = ((V1 × 0.01 × 1.4)/V2) × 1.2878, where V1 is the volume (mL) of HCl used for the titration, 0.01 is the HCl normality, V2 is the volume of the fermented medium used (mL), and 1.2878 is the nitrogen–ammonium conversion factor. Lactate is an intermediate product of the fermentation of fibre polysaccharides [30]. Lactate production was measured using a commercial kit (MAK 329, Sigma-Aldrich, St. Louis, MO, USA), following the manufacturer’s procedure, and absorbance at 565 nm was recorded. The results are expressed in mM. The antioxidant capacity of the aliquots of the fermentation medium was quantified using the FRAP method according to the procedure mentioned above.

### 2.12. Statistical Analysis

The analysis of the BSG was conducted using nine batches of samples, whereas the analysis of the BSG flour was conducted in triplicate. The data were processed using the SPSS 24.0 software package (LEAD Technologies, Inc., Chicago, IL, USA), and the results were expressed as the mean and standard deviation. For the in vitro fermentation assay, two-way analysis of variance (ANOVA) was carried out to analyse the differences in the production of SCFAs, ammonium, and lactate according to fermentation time and substrate. Pearson’s correlation analysis was also used to determine the relationships among the different parameters of the BSG flour.

### 2.13. Microbiota Profiling by Targeted 16S rRNA Amplicon Sequencing

One mL of each fermentation aliquot (time 0 h, 24 h, and 48 h) was centrifuged at 16,110 g, and the supernatants were removed. The precipitates were used to extract DNA with the MagMAX Microbiome Ultra Nucleic Acid Isolation Kit (A42358, Applied Biosystems) on the KingFisher robot (Thermo Fisher Scientific, Waltham, MA, USA). Total DNA concentration was measured using a Qubit^®^ 2.0 Fluorometer (Life Technology, Carisbad, LA, USA) and normalized to 5 ng/μL for amplification. The V3–V4 region of the bacterial 16S rRNA gene was amplified using the primer pair 341F/805R, following the Illumina 16S metagenomic sequencing library preparation protocol. Library preparation and sequencing were performed by the Molecular Biology Laboratory (ACTI, University of Murcia) on an Illumina NextSeq 1000/2000 platform to generate paired-end reads. During sequencing, three samples corresponding to previously characterized microbial communities were included as positive controls or mock communities. For the negative control, the same protocol described by Illumina was followed, using sterile molecular-grade water as the template instead of DNA. In the final step of library preparation, this control was quantified using Qubit, and no measurable DNA concentration was detected, indicating that no relevant environmental contamination occurred during the process.

Data were processed using nf-core/ampliseq version 2.14.0 [31] from the nf-core collection of workflows [32]. Data quality was evaluated with FastQC [33] and summarized with MultiQC [34]. Cutadapt [35] trimmed primers, and all untrimmed sequences were discarded. Sequences that did not contain primer sequences were considered artifacts. Less than 3.8% of sequences were discarded per sample, and a mean of 96.6% per sample passed filtering, being a 99.64% of the sequences assigned to bacteria. Adapter- and primer-free sequences were processed sample-wise (independently) with DADA2 [36]. The amplicon sequence variant (ASV) count table contained a total of 2,061,835 reads after filtering, ranging from 36,654 to 127,827 reads per sample. Taxonomic classification was performed with DADA2 and the database ‘Silva 138.2 prokaryotic SSU’ [37], and the results were expressed as relative abundance at different levels and for specific bacteria. Indices of α-diversity were measured using the Fisher index, with Mann–Whitney tests followed by Benjamini–Hochberg correction for *p*-value adjustment. β-diversity was assessed using Unweighted UniFrac distances and Principal Coordinate Analysis (PCoA). Permutational Multivariate Analysis of Variance (PERMANOVA) was used to determine differences in the overall microbial community structure. Analysis was performed using MicrobiomeAnalyst 2.0 [38].

## 3. Results and Discussion

### 3.1. Proximate Composition, Total (Poly)phenolic Compound Content and Antioxidant Capacity of Fresh BSG and BSG Flour

Due to the high moisture content of BSG, its valorization as a food ingredient requires conversion into a dry product, such as BSG flour, to facilitate handling in the food industry, maintain its nutritional composition, and ensure food safety. In this study, fresh BSG was obtained from a brewery as wet samples and was dehydrated to obtain BSG flour. As shown in Table 1, the drying process concentrated nutrients in the flour relative to fresh BSG, and the values obtained for both fresh BSG and BSG flour were consistent with previously reported data [2]. The caloric values were 186 kcal/100 g and 419 kcal/100 g for fresh BSG and BSG flour, respectively.

The compositional profile of BSG flour is characterized by a high carbohydrate content, largely associated with its dietary fibre fraction, in which insoluble dietary fibre predominates (93%). The TDF content in BSG flour (45.06%) was comparable to that of wheat bran (42.8%) [39], a widely used ingredient in the food industry. Due to its IDF content, BSG flour can exert several beneficial effects on gut and metabolic health, acting as a bulking and laxative agent that reduces intestinal transit time and decreases glucose and fat absorption. In addition, IDF has been shown to positively regulate postprandial insulin and has been associated with a reduced risk of diabetes, as well as beneficial effects on metabolic and inflammatory markers related to metabolic syndrome, and direct or indirect effects on the gut microbiota [40].

The total (poly)phenolic compound content was quantified in two fractions: a fraction extracted with an aqueous organic solvent (EPPs) and a fraction containing hydrolysable compounds, as well as those linked to dietary fibre and proteins, extracted using acidic hydrolysis (NEPPs) [41]. The NEPPs fraction was higher than the EPPs fraction, and TPC was calculated as the sum of both (Table 1).

Overall, the total (poly)phenolic content in BSG flour fell within the range reported in the literature, being slightly higher than the 7–10 mg GAE/g reported by Bravi et al. [42] but lower than the values described by Moreira for BSG derived from pilsner-type malts (20 mg GAE/g); lower levels have also been reported in dark malts [43]. These differences may be attributed to factors such as barley variety, malting conditions, brewing processes, and the extraction methodology used, particularly because some methods do not include the analysis of non-extractable (poly)phenols, which are linked to the polysaccharides in dietary fibre. It should be noted that NEPPs, in addition to providing antioxidant effects, also contribute to the prebiotic effect of dietary fibre because they can resist digestion in the small intestine and reach the colon, where they can be used as substrates by the colonic microbiota, generating various catabolites with beneficial effects for human health [6,44].

The antioxidant capacity of fresh BSG and BSG flour was analyzed using the FRAP method (Table 1). Compared with previous studies, the values obtained for BSG flour were lower than those reported by Bravi et al. (30 to 40 µmol TE/g) [42], which may be due to differences in raw materials (malt) or malting processes.

### 3.2. Amino Acid Content and Mineral Composition of BSG Flour

Table 2 shows the essential and non-essential amino acid contents, expressed in mg/g and as a percentage of the total protein content in the BSG flour, as well as the mineral content and the percentage of the DRV. More than 60% of the amino acids were essential. In terms of their contribution to total protein content, the predominant ones were leucine, isoleucine, and valine. These amino acids are known as branched-chain amino acids (BCAAs) and have been studied for their role in muscle recovery in long-duration sports as well as in the recovery of liver and muscle function in chronic disease [45]. The predominant non-essential amino acids were proline, glutamate, and alanine, followed by glycine.

In addition, Table 2 shows the mineral content of BSG, expressed in mg/100 g, as well as the percentage of the dietary reference values (DRVs) for consumers aged 30 to 70 years, according to the European Food Safety Authority (EFSA) recommendations. In general, 100 g of BSG flour covers more than 50% of the DRVs for most minerals, except calcium, although its level is higher than that found in other commonly consumed whole grain flours [39]. Compared with other whole wheat flours, such as whole wheat flour, oat bran, or dark rye flour, BSG contains high levels of zinc and iron [39]. BSG flour can be considered a good source of iron and manganese, reaching almost 100% of their DRVs.

The silicon content in the BSG flour had a mean value of 17 mg/100 g. Despite the absence of a defined DRV for silicon, the BSG flour can be considered a food product rich in silicon, since its level is greater than 10 mg/100 g [46] and higher than that in dry dates (16 mg/100 g) or other cereals like oat bran (10 mg/100 g), which is the main source of this mineral in the diet [47]. Adequate silicon levels are associated with increased collagen synthesis, contributing to the structural integrity of the skin, hair, and nails, as well as the arterial wall, reducing the risk of atherosclerosis. In addition, silicon also provides benefits such as reducing the bioavailability of aluminum by partially blocking its gastrointestinal absorption and thus helping prevent neurodegenerative diseases such as Alzheimer’s [48]. Beer contains 2 mg of silicon/100 g [48], and studies have shown that the benefits of beer consumption are associated with high organic silicon levels [46]. The silicon content in the BSG flour is higher than that of beer, and taking into consideration that BSG flour has been used as an ingredient in different proportions that vary from 10–30% [49], foods formulated with BSG flour may contribute to the increased dietary silicon intake, enabling people to obtain the beneficial effects of this mineral, while avoiding alcohol consumption.

### 3.3. Neutral Sugars and Uronic Acids of Dietary Fibre in BSG Flour

The TDF composition of BSG flour was analyzed for neutral sugar and uronic acid contents (Table 3). Xylose and glucose were the most abundant neutral sugars, followed by arabinose, whereas galactose, mannose, and uronic acids had mean values < 5%. In addition, rhamnose was not detected in BSG flour, consistent with the low levels typically found in cereal pectin [50], suggesting that the remaining pectin consists of weakly branched pectic polysaccharides. Xylose is a major component of insoluble hemicelluloses, and glucose is a major component of cellulose and hemicellulose, both of which contribute to the high IDF content in the sample.

The proportions of polysaccharides within the total dietary fibre (TDF) were estimated from the relative amounts of neutral sugars and uronic acids (Table 3), using the formulas described in Section 2.5.

The high percentages of xylose and arabinose suggest that BSG flour also contains a high proportion of arabinoxylans, as reported previously [2,51]. On the other hand, the low contribution of mannose to the hemicellulose structure, indicated by the low mannose/xylose ratio [52], suggests that the hemicellulose in BSG flour is mostly insoluble, a finding also reported by other authors [53]. Arabinoxylans originate in the cell walls of barley and remain in the BSG after the malting process. In addition, ingredients containing arabinoxylans can be treated with hydrolytic methods (such as hydrothermal and enzymatic methods) to obtain xylose and arabinose, which have positive effects on human health. Both compounds can stimulate the growth and/or activity of beneficial gut bacteria (especially those from the genera *Lactobacillus* and *Bifidobacterium*), leading to the production of SCFAs, while inhibiting the growth of pathogenic bacteria [51].

There is limited information in the literature on the uronic acid content of BSG. Langenaeken et al. [54] reported that the uronic acid content of barley endosperm increased from 5% to 33% during malting, due to carbohydrate hydrolysis. Some authors reported low levels of uronic acids, mannose, and galactose in BSG (ranging from 1.5 to 3.5%), which are components of pectin and hemicellulose [55]. In this study, the levels of these components were within the previously reported ranges. The pectin content of the BSG flour had a mean value < 24%, making it the least abundant non-starch polysaccharide in the dietary fibre, and was estimated based on the galacturonic acid content, the main structural component of its backbone. However, the colorimetric method used does not distinguish between uronic acids derived from pectin and those present in other polysaccharides, such as hemicelluloses; therefore, the pectin content should be considered an approximation. In addition, the formulas used do not account for uronic acids in hemicellulose, which may lead to a slight overestimation of pectin. Nevertheless, the contribution of uronic acids from hemicellulose in barley is generally low, and the hemicellulose fraction of BSG is mainly composed of xylose, as reported for other cereal-derived by-products [56]. These results should therefore be interpreted as an estimation of the relative distribution of polysaccharide fractions rather than as exact quantitative values.

### 3.4. Techno-Functional Properties of BSG Flour

The techno-functional properties of the BSG flour are presented in Table 4, providing information on its potential use as an ingredient in the food industry (FAC, WRC, and SWC) and its physiological effects on human health (OP and GDRI).

The mean FAC value for BSG flour was 2.4 g/g, lower than values reported in the scientific literature for wheat by-products and bagasse [57,58] but higher than those for by-products from fruits, vegetables, or seaweeds [59]. The high FAC of BSG flour may help prevent fat loss during cooking, improve product shelf life, and be useful in bakery production. Moreover, adding BSG flour could reduce oil/fat absorption during digestion [60] by increasing its excretion with the fibre. The mean WRC and SWC values were 7.72 g water/g and 9.09 mL water/g, respectively, higher than data reported by other authors for malt bagasse [61]. These properties indicate the fibre’s ability to retain water in its structure, which has been shown to increase fecal volume and intestinal mobility, helping to prevent intestinal diseases [40]. Some technological processes used in flour production, such as milling, can increase the hydration properties (WRC and SWC) and FAC of novel ingredients by reducing particle size, thereby increasing the surface area and total pore volume [62]. The OP of BSG flour was evaluated as an in vitro indicator of potential problems associated with diarrhea after ingesting a large amount of dietary fibre. The OP value we obtained (291 mosM/Kg NaCl) was like that of dietary fibre in tomato peels [59] and raspberry dietary fibre [28]. This value suggests that diarrhoea problems are unlikely after the consumption of BSG flour, since its osmotic pressure is lower than the physiological osmotic pressure. Due to the high IDF content, the GDRI was higher at 15 min (36%), then decreased to 3.5% at 30 min, suggesting that BSG flour can reduce glucose absorption, mainly by trapping glucose molecules in the network formed by the IDF components [59].

### 3.5. (Poly)phenolic Compounds and Antioxidant Capacity of NNPs and EPPs Fractions

The (poly)phenolic compounds in the BSG flour were investigated using the Folin method (Table 1) and HPLC-DAD [63] (Table 5). As mentioned above, NEPPs accounted for the highest proportion of (poly)phenolic compounds and represented the fraction bound to polysaccharides of TDF through hydrogen bonds and Van der Waals forces [64]. In terms of individual compounds, hydroxycinnamic acid derivatives, including ferulic acid and p-coumaric acid, were identified as the major phenolics, while flavonols (quercetin and kaempferol derivatives) were present in lower amounts in the EPPs fraction. In the NEPPs fraction, only hydroxycinnamic acid derivatives were detected, mainly ferulic acid dimers and syringic acid. The concentration of all phenolic compounds in both fractions (EPPs + NEPPs) reached 1273 µg/g of BSG.

Our results align with the scientific literature, as other authors have reported that BSG contains hydroxycinnamic acids, including ferulic acid (359 to 1948 µg/g dry weight), p-coumaric acid (79 to 794 µg/g dry weight), caffeic acid (<19 µg/g dry weight), and sinapic acid, along with minor amounts of hydroxybenzoic acid, flavonols, and proanthocyanidins [55]. In addition, Birsan et al. [65] found small amounts of catechin, 4-hydroxybenzoic acid, sinapic acid, syringic acid, and protocatechuic acid in BSG, with total free and cell structure-bound (poly)phenolic compound content ranging from 550 to 2741 µg/g dry weight of BSG. The (poly)phenol content and profile of BSG depend on the genotype and type of malt used, the growing environment, and the production process. Studies have shown that most bioactive phenolic acids are present in a bound form in BSG [66].

The antioxidant capacity measured by the FRAP method was markedly higher in the NEPPs fraction (20.75 µmol Trolox/g) than in the EPPs fraction (1 µmol Trolox/g), indicating a greater contribution of bound (poly)phenols to the overall antioxidant capacity of BSG. However, from a nutritional and physiological perspective, this effect can only be realised when these bound (poly)phenols are released from the food matrix during gut fermentation of non-starch polysaccharides, thereby contributing to the antioxidant status of the intestinal lumen.

### 3.6. Prebiotic Effect of BSG Flour

**Figure 1:**
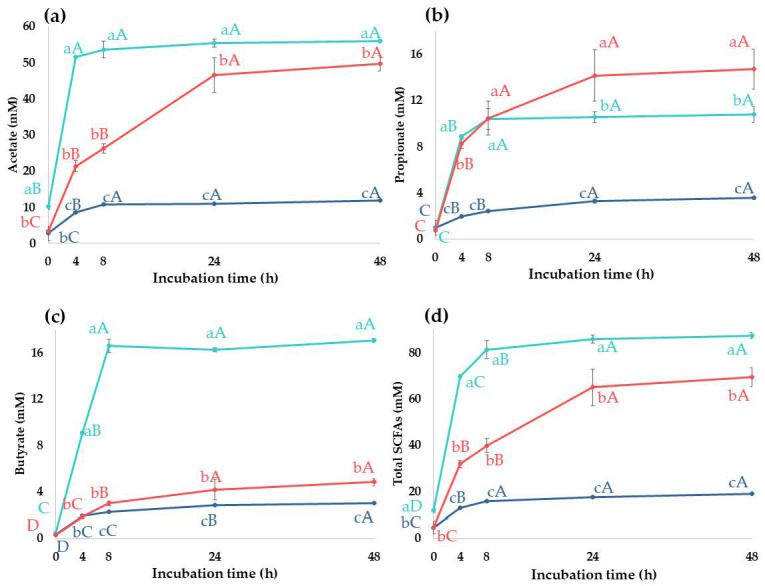
SCFAs production (acetate (**a**), propionate (**b**), butyrate (**c**) and total SCFAs (**d**)) (mM) during *in vitro* fermentation of bagasse flour samples at 0, 4, 8, 24 and 48 h of incubation. Bagasse flour (●BSG), positive control (●C+) and negative control (●C−). Values are expressed as mean ± SD (n = 3). Different letters indicate significant differences according to two-way ANOVA. Lowercase letters (a–c) indicate significant differences (*p* < 0.05) between samples at the same time point. Capital letters (A–D) indicate significant differences between fermentation time points for the same sample.

**Figure 2:**
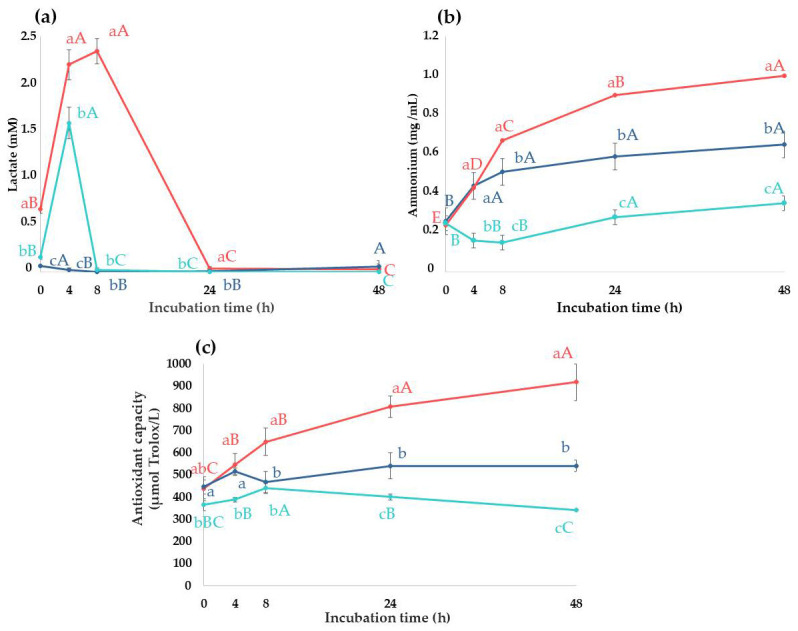
Lactate (**a**) (mM), ammonium (**b**) (mg/mL of faecal slurry) and antioxidant capacity (**c**) (μmol Trolox/L) during in vitro fermentation of bagasse flour samples at 0, 4, 8, 24 and 48 h of incubation. Bagasse flour (●BSG), positive control (●C+) and negative control (●C−). Values are expressed as mean ± SD (n = 3). Different letters indicate significant differences according to two-way ANOVA. Lowercase letters (a–c) indicate significant differences (*p* < 0.05) between samples at the same time point. Capital letters (A–E) indicate significant differences between fermentation time points for the same sample.

To assess the prebiotic effect of BSG flour, an in vitro fermentation model was used, as this methodology can be used to select potential ingredients with prebiotic effects and allows the identification and quantification of resulting fermentation products, especially SCFAs, as well as the analysis of microbiota enzymatic activity and harmful compounds, and the analysis of microbiota genera/species [67]. In this research, the prebiotic effect of BSG flour was determined according to gut microbiota activity by measuring SCFAs, which are beneficial compounds for human health (Figure 1a–d). Nowadays, SCFAs are recognized to play a vital role in both healthy and ill hosts by regulating metabolism, immune function, and inflammation, and have therapeutic effects on gastrointestinal and neurological disorders, as well as antitumor effects [68]. In addition, lactate and ammonium, as end products of gut microbiota activity, as well as changes in the antioxidant capacity in the medium of in vitro fecal fermentation, were also investigated (Figure 2a–c), with the aim of ascertaining the release of (poly)phenols from the food matrix. The experimental samples (BSG flour) were assayed in parallel with a positive control (C+) that included glucose, allowing the viability of the bacteria to be assessed, and a negative control (C−) in which no substrate was added. All parameters were measured at the beginning (0 h) and during the fermentation assay at 4, 8, 24, and 48 h.

Overall, the total SCFA content increased significantly during fermentation in the C+ due to rapid glucose metabolism by the microbiota. The C− also showed a significant increase in SCFAs over time, but levels were lower because no fermentation substrate was present. Significant differences were observed in SCFA production during BSG flour fermentation compared with both controls, and the ANOVA showed that both factors (fermentation time and experimental conditions) significantly influenced (*p* < 0.05) the content of major and minor SCFAs. The total SCFA content increased significantly during the first 24 h of in vitro faecal fermentation of BSG and then remained constant between 24 and 48 h (Figure 1d). At time 0, the total SCFA content was 4.6 mM, which increased to 64.5 mM after 48 h. Similar fermentation kinetics were observed in other studies evaluating these metabolites during in vitro faecal fermentation of different cereal brans; they reported total SCFA contents of 20 and 40 mM after 48 h [69]. In addition, our results are similar to those of an in vitro fermentation study using pre-treated oat bran (50 to 70 mM), arabinoxylan from rice bran (30 to 60 mM) [70,71], and arabinoxylan from BSG (10 to 59 mM) [51].

Regarding relative abundance, it has been reported that after in vitro fermentation of non-starch polysaccharides, the proportions of SCFAs are approximately 60% acetate, 23% propionate, and 17% butyrate [30]. In this study, acetate was the predominant SCFA (72% of total SCFA content), followed by propionate (21%) and butyrate (7%), indicating a higher proportion of acetate relative to butyrate. The minor SCFAs did not show a significant change during the fermentation process and remained constant from 0 to 48 h, with a mean value of <1 mM.

The production of acetate and propionate increased significantly during the first 24 h of fermentation, due to the high IDF content of the BSG flour, particularly cellulose, hemicellulose, and arabinoxylans, a pattern also observed by other authors [51,72]. In contrast, butyrate, mainly produced by *Eubacteria*, *Clostridia*, and *Fusobacteria* through saccharolytic and amino acid fermentation [67] and largely associated with the presence of fermentable glucose or soluble dietary fibre (SDF) [73], was produced in greater quantities in the positive control (C+) and at very low levels during the fermentation of BSG flour. This result could be explained by the low SDF content in BSG, which represents only 7% of the TDF content. This behaviour is consistent with the findings of Lynch et al. [51], who reported that BSG-derived arabinoxylans exert a predominantly bifidogenic and propionogenic prebiotic effect, characterised by increased acetate and propionate production and only moderate butyrate formation.

In addition to fiber composition, factors such as particle size and the substrate’s physical structure may influence metabolite formation through fiber–microbiota interactions, with smaller particle sizes (100–150 µm) associated with higher acetate and propionate production [69]. In this study, milling dried BSG produced small particle sizes, which may have contributed to the observed SCFA profile.

Furthermore, in vitro batch fermentation models have limitations because they cannot fully replicate the physiological cross-feeding mechanisms that occur in vivo. In particular, acetate produced by *bifidobacteria* can be converted to butyrate by butyrogenic species such as *Eubacterium rectale*; therefore, butyrate production may be underestimated under in vitro conditions [74].

Lactate, an intermediate product of fermentation, was also analyzed throughout fermentation (Figure 2a). Production followed a trend similar to that of the SCFAs during the first hours of fermentation but decreased thereafter. However, BSG flour showed intense production during the first 8 h of fermentation compared with the control samples. As fermentation progressed, lactate decreased significantly, while propionate increased in parallel (Figure 1b). BSG flour showed the highest production of propionate, which can be synthesized from lactate [75]. The increase in acetate and lactate production after BSG flour fermentation could be related to the saccharolytic activity of *bifidobacteria* [76], suggesting a positive effect on modulating the growth of the gut microbiota.

Ammonium originates from the catabolism of dietary proteins and amino acids when carbohydrate levels are low in the intestinal lumen [30]. Protein degradation in the colon occurs in several steps and involves different bacteria, leading to the formation of metabolites (amines, ammonia, sulfur compounds, and mercaptans) that, due to their toxic effects, may affect host health [30]. Among amino acids, glutamate and glutamine are the most prominent contributors to nitrogen and ammonia metabolism, through mechanisms in which bacteria with urease activity, abundant in the colon, are involved [77]

In our study, ammonium production (Figure 2b) in the control samples was primarily driven by bacterial consumption of tryptone, which served as a fermentation substrate and contained nitrogen as its main component. However, there were differences between the two controls: in the negative control, bacteria consumed tryptone directly because no other carbon source was added to the fermentation medium. In contrast, in the positive control, the presence of glucose initially suppressed tryptone consumption. After 8 h of fermentation, when glucose was completely consumed, tryptone consumption resumed. Fermentation with BSG flour (Figure 2b) showed a significant increase in ammonium production over time, reaching values < 1 mg/mL (<0.05 mM) at 48 h, mainly due to the high protein content in this product (24.41%, Table 1). Although ammonia is considered a harmful metabolite that has been correlated with inflammatory pathologies in the large intestine, its formation is related to the presence of protein, amino acids, and phenolic compounds, which are present in the BSG sample as a complex food. However, different studies have shown that providing plant-based proteins to the gut microbiota leads to an improvement in the gut barrier and increases SCFA levels and the abundance of *Bifidobacterium*, *Lactobacillus*, and *Enterococcus*, while decreasing the abundance of *Bacteroides*, *Clostridium perfringens*, and *Escherichia coli* [9].

Regarding the changes in antioxidant capacity in the medium during fermentation (Figure 2c), a significant increase is observed after 8 h of BSG flour fermentation, due to the saccharolytic activity of the microbiota and the release of (poly)phenols from the food matrix. This result shows that BSG not only contributes to improving the metabolites of the gut microbiota but also could enhance the antioxidant environment of the intestinal lumen, since there is an increase in the ferric reducing ability, mainly due to the release of (poly)phenols bound to non-starch polysaccharides.

**Figure 3:**
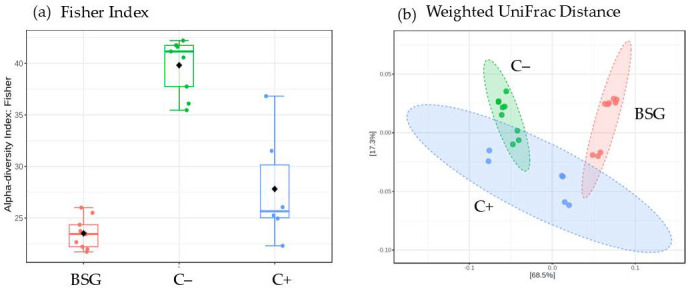
α-diversity (Fisher index) and β-diversity (PCoA based on Weighted UniFrac Distance) of gut microbiota during in vitro fermentation of BSG flour (BSG), negative control (C−) and positive control (C+).

To confirm changes in the gut microbiota, α-diversity and β-diversity were measured (Figure 3a,b). α-diversity was evaluated using the Fisher index to assess microbial diversity within samples (BSG flour, C+ and C− groups). The Fisher index estimates species richness, with higher values indicating greater microbial diversity and more taxa within the community, whereas lower values suggest reduced richness and a less complex microbial structure. The Fisher index differed significantly between BSG flour and C− (FDR *p* < 0.001), whereas no significant differences were observed between BSG flour and C+ (FDR *p* = 0.1). Therefore, α-diversity reflected changes in microbial community richness associated with the experimental conditions, showing a reduction in microbial richness in BSG flour, which could be explained by the dominance of specific taxa. The decrease in the Fisher index observed in the BSG group could be due to the selective effect of the complex fibers present in this substrate (mainly IDF and a high polyphenol content). Several studies have shown that certain dietary fibers favor specialized microorganisms capable of metabolizing complex polysaccharides, thereby reducing the overall richness and diversity of the microbiota [78]. Likewise, the physicochemical and structural properties of plant fibers can generate specific ecological niches that limit the growth of other bacterial taxa. For this reason, analyzing the abundance of specific taxa is important for understanding the biological relevance of BSG to the colonic microbiota. Additionally, β-diversity was represented by PCoA based on Weighted Unifrac Distance at the feature level (Figure 3b). At time 0 h, C+ and C− were close in the PCoA, showing a similar microbiota profile, whereas BSG flour was farther away due to the substrate’s initial microbial characteristics. The observed clustering pattern was confirmed by PERMANOVA analysis, indicating that microbial community composition differed significantly among experimental groups (R^2^ = 0.69, FDR = 0.001) (Figure 3b). Hence, 69% of the variation was explained by the experimental condition of fermentation (BSG flour, glucose (C+) and only faeces (C−)).

**Figure 4:**
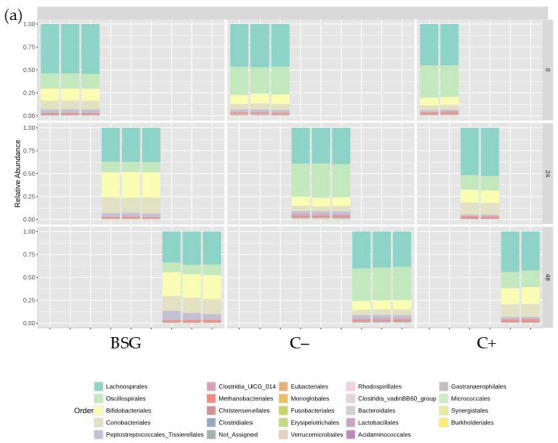

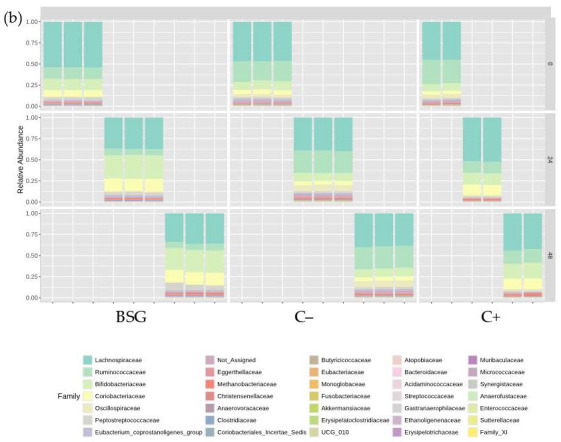

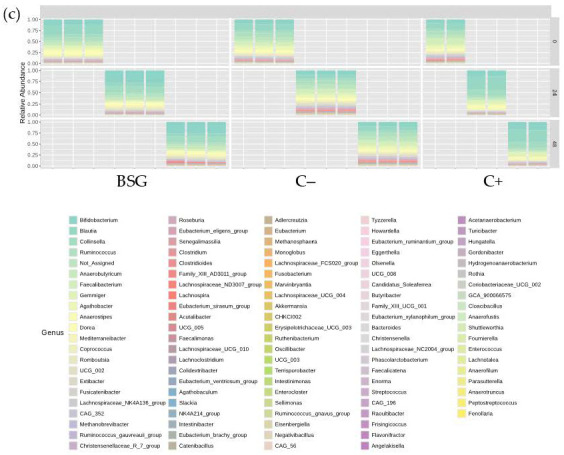
Relative abundance for the taxonomic classification at order (**a**), family (**b**) and genus (**c**) levels, observed during in vitro fermentation of BSG flour (BSG), negative control (C−) and positive control (C+). Values are shown at the beginning (0 h) and after 24 h and 48 h of in vitro fermentation.

The taxonomic composition and changes in the gut microbiota, expressed as relative abundance at the order, family, and genus levels, are shown in Figure 4a–c. Taxonomic analysis revealed differences in microbial composition across several hierarchical levels, including order, family, and genus. At the order level (Figure 4a), shifts in the relative abundance of dominant microbial groups suggest that the experimental condition may influence the broader structure of the gut microbiota. 13 microbial groups showed significant differences during in vitro fermentation of BSG flour, highlighting increases in *Bifidobacteriales* (significant differences with both controls) and decreases in *Oscillospira* and *Lachnospirales*. A similar profile was observed in the main groups of C+, whereas in C−, the profile of the gut microbiota remained constant during fermentation. These patterns were further reflected at the family level (Figure 4b), where specific families showed differential relative abundances across samples, indicating potential ecological adaptations or functional changes within the microbiota. There was a marked reduction in *Lachnospiraceae* and *Ruminococcaceae,* and an increase in *Bifidobacteriaceae* and *Coriobacteriaceae* in BSG flour, showing significant differences with C−. This tendency was also observed in C+, but no significant differences were observed at 48 h for the families *Bifidobacteriaceae* and *Coriobacteriaceae* compared with BSG flour. At the genus level (Figure 4c), the observed variations highlighted more specific microbial signatures associated with the in vitro fermentation of BSG flour, suggesting that a particular genus may contribute to the observed restructuring of the gut microbiota. In this context, the relative abundance of *Bifidobacterium* increased significantly after fermentation of BSG flour compared with the controls, suggesting that the gut microbiota undergoes compositional changes that may reflect underlying ecological or host-related processes. In particular, changes in *Bifidobacterium* suggest that the non-starch polysaccharides and other macronutrients (proteins and polyphenols) in BSG flour positively affect the gut microbiota, potentially exerting a prebiotic effect. Many plant glycans (such as non-starch polysaccharides from TDF) are believed to stimulate the metabolism and growth of specific bifidobacterial species and, for this reason, are considered prebiotic [79]. *Bifidobacterium* species are associated with various beneficial effects for the host, such as pathogen protection, including production of acetate to protect against enteropathogenic infection [80], sequestration of iron at the detriment of gut pathogens [81], immune modulation through exopolysaccharide production [82], alleviation of irritable bowel syndrome [83], and reducing the contracting rotaviral diarrhea [84].

**Figure 5:**
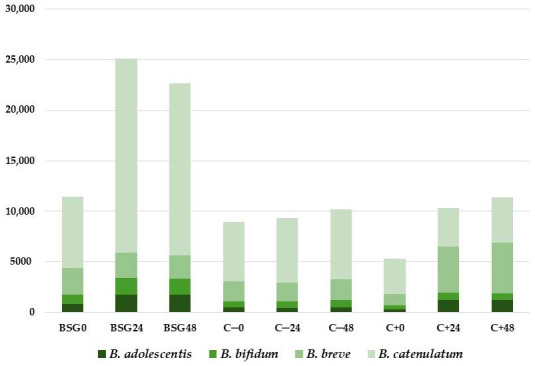
Changes in absolute abundance (ASV) of *Bifidobacterium* species during in vitro fermentation of BSG flour (BSG), negative control (C−), and positive control (C+). Values are shown at the beginning (0 h) and after 24 h and 48 h of in vitro fermentation.

Species-level analysis highlighted that four *Bifidobacterium* species (*B. catenulatum*, *B. breve*, *B. bifidum*, and *B. adolescentis)* contributed to the observed microbial community shifts. The significant increase (*p* < 0.05) in the absolute abundance of these Bifidobacterium species after fermentation of the BSG flour (Figure 5) provides a more refined view of its prebiotic effect. These increases were also observed in control samples (C− and C+), but at lower absolute abundance. In addition, significant differences (*p* < 0.05) were observed in the abundance of the four species at 24 h and 48 h across the three samples. *B. catenulatum* showed the greatest shift in the BSG flour, and although the current scientific evidence is limited and often strain-specific, many of its proposed health benefits are inferred from the genus. These findings suggest that the interaction of BSG flour with the gut microbiota could have important implications for host–microbiota interactions and overall intestinal health.

Although gut bifidobacteria cannot metabolize insoluble complex plant polysaccharides, such as BSG flour with 41% of IDF (Table 1), this microbial genus may be able to utilize specific components and/or side chains of non-starch polysaccharides (for example, in the hemicellulose and arabinoxylan chains). In addition, these polysaccharides can be degraded by specific species, so-called keystone species. Examples of keystone species include different *Bacteroides* species, which are capable of degrading polysaccharides and releasing oligosaccharides that may then become available as substrate for other gut commensals, such as *bifidobacteria* [79].

Given that the definition of a prebiotic refers to a substrate selectively utilized by host microorganisms, conferring a health benefit [85], we can consider that BSG has an indirect prebiotic effect, as it promotes the growth of *Bifidobacterium* species (Figure 5) and leads to a significant increase in SCFAs. In particular, *bifidobacteria* produce acetate and lactate as their primary fermentation end-products, as described above (Figure 1 and Figure 2), whereas butyrate and propionate are produced indirectly. Other gut bacteria use acetate and lactate through metabolic cross-feeding to produce butyrate and propionate [76]. Other authors have reported a prebiotic effect of BSG arabinoxylans and a bifidogenic effect (a significant increase in acetate and propionate levels) after *Lactobacillus* stimulation [51]. In addition, increased acetate and lactate levels are linked to the stimulation of *bifidobacteria* [86], whereas propionate production is linked to *Bacteroides* [87]. These findings align with our results, and hence, the dietary fiber in BSG flour is recognized as a key driver of SCFA production with potential prebiotic effects by increasing acetate, propionate, and butyrate levels, which play important roles in host homeostasis and the regulation of bacterial community dynamics.

Moreover, the metabolism of glycans from TDF, along with the release of NEPPs during in vitro fermentation, could benefit gut health, since (poly)phenols can promote the growth of beneficial bacteria and inhibit harmful ones [9]. Kuhn et al. [88] reported that grape proanthocyanidins, considered NEPPs, can reduce microbiota dysbiosis and increase *Akkermansia muciniphila* in rats, maintaining antioxidant capacity at 40% in the feces after 24 h. In this study, changes in antioxidant capacity during BSG flour fermentation could be linked to the release and breakdown of NEPPs, which may, at least partially, reduce ROS and restore a more favorable redox potential in the gut, thereby promoting a healthier gut microbial environment [88]. In addition, a significant increase in the abundance of *Slackia piriformis* was observed during BSG flour fermentation compared with the controls. The genus *Slackia* comprises anaerobic bacteria commonly found in the human gut microbiota that metabolize (poly)phenols and isoflavones, transforming them into bioactive molecules with diverse metabolic activities that can influence antioxidant capacity, hormone-like effects, and gut microbial balance. *Slackia* bacteria may also help regulate the composition of the intestinal microbiome and participate in processes that protect the gut environment, potentially contributing to improved digestion and overall metabolic health [89]. Considering the (poly)phenol profile of BSG flour, *Slackia piriformis* could participate in the reduction of hydroxycinnamic acids to phenyl propionic acid derivatives [90], but the gut (poly)phenol metabolites were not analyzed in this research.

## 4. Conclusions

BSG flour showed high nutritional value, characterized by a high content of dietary fibre (hemicellulose and arabinoxylans), together with proteins, non-extractable (poly)phenols, and silicon, as well as favourable techno-functional properties such as fat absorption and water retention capacities. The in vitro fermentation indicates that BSG flour plays an important role in stimulating gut microbial growth and metabolic activity, leading to increased production of short-chain fatty acids, particularly acetate and propionate. Microbiota profiling further revealed compositional changes during fermentation, including an increased relative abundance of *Bifidobacterium* species, suggesting a potential bifidogenic effect.

Overall, these findings support the potential of BSG flour for use as a sustainable ingredient for developing fibre-rich foods with microbiota-modulating properties. The valorisation of this by-product is consistent with current strategies aimed at reducing food waste and promoting a circular economy, including initiatives such as the United Nations Sustainable Development Goals, which encourage the reuse of agri-food by-products in higher-value applications. However, further in vivo studies are required to confirm these effects in human subjects.

## Figures and Tables

**Table 1:** Proximate composition, antioxidant capacity (FRAP), and phenolic compounds: extractable (EPPs), non-extractable (NEPPs), and total phenolic compound (TPC) contents of fresh BSG and BSG flour ^1^.

Proximate Composition	BSG	BSG Flour
Moisture (%)	74.70 ± 2.40	1.43 ± 0.11
Protein (%)	7.27 ± 0.89	24.41 ± 0.10
Fat (%)	0.10 ± 0.02	8.35 ± 0.42
Ash (%)	0.96 ± 0.09	4.31 ± 0.29
Total Carbohydrate * (%)	16.97 ± 1.63	61.50 ± 0.54
Total Dietary Fibre (TDF) (%)	12.16 ± 1.08	45.06 ± 1.66
Insoluble Dietary Fibre (IDF) (%)	11.31 ± 1.00	41.9 ± 1.52
Soluble Dietary Fibre (SDF) (%)	0.85 ± 0.08	3.15 ± 0.12
Caloric Value (kcal 100 g)	186.12 ± 9.28	418.73 ± 1.62
**Phenolic compounds**		
EPPs (mg GAE **/g)	0.25 ± 0.02	1.43 ± 0.11
NEPPs (mg GAE/g)	1.21 ± 0.04	11.50 ± 0.30
TPCs *** (mg GAE/g)	1.46 ± 0.03	12.90 ± 0.38
**Antioxidant capacity**		
FRAP **** (µmol TE/g)	6.90 ± 0.03	21.77 ± 0.70

^1^ Data are expressed as the mean ± standard deviation (*n* = 9 for BSG and n = 3 for BSG flour). * The total carbohydrate content includes the total dietary fibre (TDF) content. ** GAE: gallic acid equivalent; *** TPC: total phenolic compound; **** FRAP: ferric reducing antioxidant power.

**Table 2:** Contents of essential and non-essential amino acids (expressed as mg/g of protein), the percentage of total protein for each amino acid, mineral content, and the percentage of the Dietary Reference Value (DRV) for each mineral in the BSG flour ^1^.

Essential Amino Acids	mg/g	% of Total Protein
Histidine	4.33 ± 0.05	1.80
Isoleucine	29.5 ± 1.91	12.27
Leucine	45.78 ± 1.52	19.04
Lysine	4.3 ± 0.01	1.79
Phenylalanine	5.67 ± 0.10	2.36
Threonine	2.10 ± 0.13	0.90
Tryptophan	1.98 ± 0.02	0.87
Valine	14.15 ± 0.09	5.88
Methionine	ND	ND
Cysteine	ND	ND
4-OH Proline	ND	ND
TOTAL	107.81	44.91
**Non-essential amino acids**	**mg/g**	**% of total protein**
Alanine	10.82 ± 0.22	4.50
Arginine	1.55 ± 0.08	0.64
Asparagine	ND	ND
Aspartate	4.58 ± 0.48	1.91
Glutamate	11.14 ± 0.12	4.63
Glutamine	ND	ND
Glycine	9.72 ± 0.29	4.04
Proline	17.51 ± 1.04	7.28
Serine	5.14 ± 0.09	2.13
Tyrosine	3.69 ± 0.03	1.54
**Minerals**	**mg/100 g**	**DRV** * **(mg/day)**
Mg	170 ± 0.01	300–350
P	490.5 ± 0.5	700
Zn	5.4 ± 0.01	8–11
Na	30 ± 0.01	<1000
Ca	220 ± 1.00	900–950
Fe	8.67 ± 0.28	9–18
Cu	0.82 ± 0.01	1–1.30
Mn	4.44 ± 0.03	3
Si	17.10 ± 1.45	-

^1^ Data are expressed as mean ± SD (*n* = 3). ND: not detected. * DRV: dietary reference value.

**Table 3:** Percentage of neutral sugars and uronic acids in the dietary fibre of BSG flour and percentage of non-starch polysaccharides ^1^.

Monosaccharides (%)	
Xylose	42.00 ± 1.12
Glucose	32.43 ± 3.29
Arabinose	17.53 ± 1.52
Galactose	2.42 ± 0.35
Mannose	1.59 ± 0.31
Uronic Acids	4.19 ± 0.33
**Non-starch polysaccharides (%)**	
Cellulose	29.19 ± 3.03
Hemicellulose	46.83 ± 0.84
Pectins	23.97 ± 2.14

^1^ Values are expressed as mean ± SD (*n* = 3).

**Table 4:** Technological (FAC, WRC and SWC) and physiological and functional (OP and GDRI) properties of BSG flour ^1^.

Properties	Mean ± SD
Fat absorption capacity (FAC) (g oil/g)	2.4 ± 0.01
Water retention capacity (WRC) (g water/g)	7.72 ± 0.15
Swelling capacity (SWC) (mL water/g)	9.09 ± 0.12
Osmotic pressure (OP) (mosM/kg ClNa)	291 ± 1.4
Glucose diffusion retardation index (GDRI) %	
15′	36.18
30′	3.51

^1^ Values are expressed as mean ± SD (*n* = 3).

**Table 5:** Concentration of (poly)phenols, determined using HPLC, and antioxidant capacity (FRAP) of extractable (EPPs) and non-extractable (NEPPs) (poly)phenolic fractions ^1^.

Extractable Fraction (EPPs)	
Total hydroxycinnamic acid derivatives content (µg/g)	354 ± 15
Total flavonols content (µg/g)	34 ± 1
FRAP * (µmol TE/g)	1.01 ± 0.09
**Non-Extractable Fraction (NEPPs)**	
Total hydroxycinnamic acid derivative content (µg/g)	886 ± 52
Total flavonols content (µg/g)	ND
FRAP (µmol TE/g)	20.75 ± 0.64
**Total (EPPs + NEPPs)**	
TPC content ** by HPLC (µg/g)	1273 ± 43
Total FRAP (µmol TE/g)	21.77 ± 0.70

^1^ Values are expressed as the mean ± SD (*n* = 3). * FRAP: ferric reducing antioxidant power; ** TPC: total phenolic compounds. ND: not detected.

## Data Availability

The data presented in this study are not publicly available due to confidentiality obligations associated with an industrial collaboration and the funding of the research by Estrella de Levante S.A.U.
